# Stable gene transfer of CCR5 and CXCR4 siRNAs by sleeping beauty transposon system to confer HIV-1 resistance

**DOI:** 10.1186/1742-6405-5-16

**Published:** 2008-07-30

**Authors:** Mayur Tamhane, Ramesh Akkina

**Affiliations:** 1 Dept. Microbiology, Immunology and Pathology, Colorado State University, Fort Collins, Colorado, 80523, USA

## Abstract

**Background:**

Thus far gene therapy strategies for HIV/AIDS have used either conventional retroviral vectors or lentiviral vectors for gene transfer. Although highly efficient, their use poses a certain degree of risk in terms of viral mediated oncogenesis. Sleeping Beauty (SB) transposon system offers a non-viral method of gene transfer to avoid this possible risk. With respect to conferring HIV resistance, stable knock down of HIV-1 coreceptors CCR5 and CXCR4 by the use of lentiviral vector delivered siRNAs has proved to be a promising strategy to protect cells from HIV-1 infection. In the current studies our aim is to evaluate the utility of SB system for stable gene transfer of CCR5 and CXCR4 siRNA genes to derive HIV resistant cells as a first step towards using this system for gene therapy.

**Results:**

Two well characterized siRNAs against the HIV-1 coreceptors CCR5 and CXCR4 were chosen based on their previous efficacy for the SB transposon gene delivery. The siRNA transgenes were incorporated individually into a modified SB transfer plasmid containing a FACS sortable red fluorescence protein (RFP) reporter and a drug selectable neomycin resistance gene. Gene transfer was achieved by co-delivery with a construct expressing a hyperactive transposase (HSB5) into the GHOST-R3/X4/R5 cell line, which expresses the major HIV receptor CD4 and and the co-receptors CCR5 and CXCR4. SB constructs expressing CCR5 or CXCR4 siRNAs were also transfected into MAGI-CCR5 or MAGI-CXCR4 cell lines, respectively. Near complete downregulation of CCR5 and CXCR4 surface expression was observed in transfected cells. During viral challenge with X4-tropic (NL4.3) or R5-tropic (BaL) HIV-1 strains, the respective transposed cells showed marked viral resistance.

**Conclusion:**

SB transposon system can be used to deliver siRNA genes for stable gene transfer. The siRNA genes against HIV-1 coreceptors CCR5 and CXCR4 are able to downregulate the respective cell surface proteins and thus confer resistance against viral infection by restricting viral entry. These studies have demonstrated for the first time the utility of the non-viral SB system in conferring stable resistance against HIV infection and paved the way for the use of this system for HIV gene therapy studies.

## Background

HIV/AIDS continues to be major public health threat with new infections on the rise. Current therapies do not completely cure the disease and there is no effective vaccine available [1,2]. A potentially rewarding approach is intracellular immunization using gene therapy strategies that protect viral susceptible cells from the infecting virus [3]. Thus far, a number of promising intracellular immunization strategies have been employed using different anti-HIV molecules that act by a variety of mechanisms. Among these, nucleic acid-based approaches using ribozymes, antisense constructs, and siRNAs have received considerable attention due to their ease of expression and their non-immunological nature [3,4]. Some of these have entered clinical trials and safety testing with encouraging results [3,4]. In these studies either conventional retroviral vectors or lentiviral vectors were used for gene transfer. Although highly efficient for stable gene transfer, use of retroviral derived vectors poses a degree of risk in terms of viral mediated oncogenesis [5]. Because of this potential risk, non-retroviral mediated gene delivery systems are being currently investigated. In this regard, Sleeping Beauty (SB) transposon system shows considerable promise [6]. This system consists of a synthetic transposon and an associated transposase which functions by a cut and paste mechanism. Gene transposition is mediated by the transposase in a two step process in which the enzyme first recognizes the short inverted/direct (IR/DR) sequences in the transposon followed by the excision of the transposon and later integration of the transposon sequences into a target DNA region with a TA-dinucleotide sequence. The SB system can be deployed either as trans-delivery system in which the transposon and transposase are delivered by independent plasmids or a cis-delivery system in which both the components are incorporated into the same plasmid [7]. Continued progress in this area has resulted in the derivation of more efficient transposases and more efficient gene delivery [8]. Many mammalian cell types have been shown to be substrates for efficient SB mediated gene transfer including mouse embryonic stem cells [9]. Thus, SB system offers a novel way of gene delivery for HIV gene therapy purposes.

With regard to effective anti-HIV genes for gene therapy, siRNAs constitute highly effective gene silencing molecules due to their target specificity and improved potency [10]. The siRNAs trigger an innate endogenous RNAi pathway for target recognition and gene silencing. Thus far, siRNAs targeted to a number of HIV genes have shown impressive gene down regulation and consequent viral inhibition both in vitro and in vivo [11-14]. Due to their high target specificity however, a high possibility exists for siRNA viral escape mutants to arise during prolonged treatment. Indeed, such generation of viral escape mutants against specific siRNAs has already been documented [15]. This possibility can be much reduced by targeting essential cellular molecules that aid in viral replication. Among the many cellular molecules shown to be involved in HIV infection and replication, the cell surface coreceptors CCR5 and CXCR4 are essential for viral entry by macrophage tropic R5 and T-cell tropic-X4 HIV respectively [16,17]. The primary HIV infection is established by R5 virus and during the later stages of disease, T-cell tropic X4 virus predominates [17,18]. In nature, a segment of the human population containing a 32-base pair deletion in the CCR5 gene, but apparently physiologically normal, was found to be resistant to infection by R5 tropic HIV-1 [17,19]. Therefore, CCR5 coreceptor is an ideal cellular target to suppress HIV infection. A number of previous studies including ours have successfully targeted both the HIV coreceptors by siRNA mediated gene silencing [12,20-22]. Down regulation of either of these coreceptors resulted in effective viral inhibition. However, retroviral derived vectors were used in these studies.

With a long range goal of developing a non-viral gene delivery of anti-HIV genes for gene therapy, here we evaluated the utility of SB transposon system to deliver siRNA genes for stable gene transfer. Two previously well characterized siRNAs against CCR5 and CXCR4 coreceptors were introduced into SB transposon. Our results show that stable cell lines can be derived that harbor and express siRNA genes with concommittent HIV resistance.

## Results

### Stable gene transfer of CXCR4 and CCR5 shRNAs by SB transposon system

To investigate the utility of SB mediated gene transfer of anti-HIV-1 coreceptor siRNAs against CCR5 and CXCR4 we used the cell lines MAGI-CCR5 and MAGI-CXCR4 that constitutively express the respective individual coreceptors in addition to a GHOST-R3/X4/R5 cell line that constitutively expresses both [23-25]. As described in the methods, the cells were transfected with the respective plasmid SB constructs. Expression of the transposed constructs was monitored by the presence of RFP fluorescence. The gene transposed cells were enriched by FACS sorting and were maintained in culture for six months to confirm stable expression of the transgenes. Expression of RFP was observed throughout the time of culture. We also evaluated cells transfected with SB constructs alone in the absence of the transposase. The RFP expression in these cells was lost within a week post transfection. In a separate set of drug selection experiments to determine the levels of gene transfer using SB system in HeLa cells, it was found that the levels of transposition were 19.5% for the RFP control (above the background 0.6% gene transfer without the transposase). The gene transfer levels for the CXCR4 siRNA and the CCR5 siRNA constructs were 10.5% and 12% respectively. To further confirm transposition mediated transgene integration in stably gene transposed cells, we analysed the genomic DNA for the presence of the respective constructs. This was achieved by PCR amplifying and sequencing the junctional region of transposon and chromosomal DNA [26]. The typical hallmark of transposition is indicated by the presence of the dinucleotide 'TA' which was found at every insertion site analysed. To determine the transposed gene location, both left and right invert/direct repeats were sequenced at the chromosomal junctions. Sequences obtained were analysed using BLASTn software. Multiple integration events were recorded which spanned a range of chromosomal regions. The integration of representative individual SB transposons into the chromosomal DNA is summarized in Table 1. GHOST-R3/X4/R5 cells transposed with the control RFP transposon showed integration in Ch 5 and 17. Cells containing CCR5 siRNA showed Ch 5 and 20 regions at the transposon integration junction, while those transgenic for CXCR4 siRNA were found in Ch 17. In case of MAGI-CCR5 cells, control RFP transposon integrated into Ch 10 and 15. The CCR5 siRNA transposed cells showed integration in Ch 12 and 20. The integration sites for MAGI-CXCR4 cells were in Ch 6 and 12 for control RFP while those for CXCR4 siRNA transposon were in Ch 5 and 7. We also analyzed the copy numbers of integrated genes in GHOST-R3/X4/R5 cells using real time PCR. Our results showed 14.3, 6.5 and 10.8 copies per cell of the RFP control, CXCR4 siRNA and CCR5 siRNA constructs (data not shown).

**Table 1:** Chromosomal integration of different SB constructs.

Cell Line	SB Construct	Chromosomal Location
GHOST-R3/X4/R5	RFP control	Ch 5q34-q35, Ch 17q25.1
	CXCR4 siRNA	Ch 17q23.3
	CCR5 siRNA	Ch 5q34-q35.1, Ch 20q13.2
MAGI-CXCR4	RFP control	Ch 6p22.3, Ch 12q14.2
	CXCR4 siRNA	Ch 5q33.1, Ch 7q31.1
MAGI-CCR5	RFP control	Ch 10p12.31, Ch 15q11
	CCR5 siRNA	Ch 12p11.2, Ch 20q13.3

### Down regulation of HIV-1 coreceptors CXCR4 and CCR5 in SB transposed siRNA transgenic cells

The above data showed that SB transposed siRNAs are stably integrated into respective cells. We next evaluated if the stably gene modified cells show the effect of siRNA mediated gene silencing. Accordingly, the transposed cells were analysed for CXCR4 or CCR5 surface expression by FACS (Figure 2). Our results showed about 94% down-regulation of CXCR4 expression and a 97% down-regulation of CCR5 in GHOST-R3/X4/R5 cells transposed with CXCR4 or CCR5 siRNAs respectively. In the MAGI-CXCR4 cell line, the CXCR4 expression was reduced by 98% by the respective siRNA, while MAGI-CCR5 cells showed a 99% reduction in CCR5 levels as a result of respective transposon mediated siRNA expression (data not shown). Cells transposed with control SB construct without siRNA insert showed no decrease in coreceptor expression with levels similar to that shown by control unmanipulated cells. The levels of coreceptor down regulation obtained with these siRNAs in SB system are similar to that seen with that delivered via lentiviral vectors (data not shown). These results confirmed the efficacy of the respective siRNAs in mediating gene silencing of the HIV-1 coreceptors.

**Figure 1:**
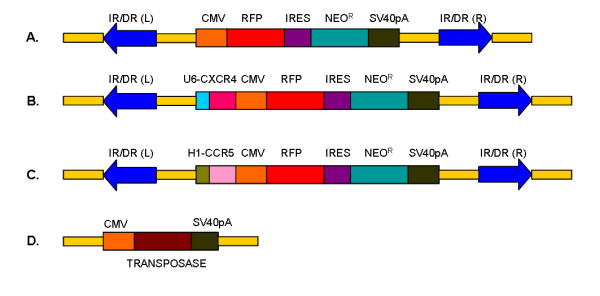
**Schematic representation of siRNA SB constructs**. A) Control SB transposon plasmid construct with Neo resistance and RFP reporter genes. RFP is driven by a CMV promoter whereas the Neo resistance is expressed via IRES. B) SB transposon construct incorporating anti-CXCR4 siRNA driven by Pol III U6 promoter. C) SB transposon construct incorporating anti-CCR5 siRNA driven by Pol III H1 promoter. D) Plasmid construct encoding the hyperactive transposase under CMV promoter.

**Figure 2:**
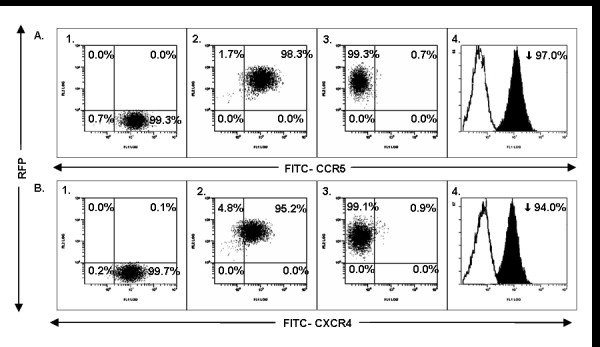
**Cell surface down regulation of CCR5 or CXCR4 coreceptors in siRNA transfected GHOST-R3/X4/R5 cells**. GHOST-R3/X4/R5 cells that constitutively express CCR5 and CXCR4 coreceptors were transfected with control RFP, CCR5 or CXCR4 siRNA constructs. RFP expressing transgenic cells were FACS sorted and cultured. To determine the down regulation of respective coreceptors, the cells were stained with respective FITC tagged antibodies and FACS analyzed. The down regulation of CCR5 coreceptor (Panel A) *was determined *by comparing CCR5 levels in untransfected (A1), control RFP transfected (A2) and CCR5 siRNA transfected (A3) cells. The CXCR4 coreceptor down regulation is shown by comparing CXCR4 levels in untransfected (B1), control RFP transfected (B2) and CXCR4 siRNA transfected (B3) cells. The percent down regulation of CCR5 (A4) or CXCR4 (B4) coreceptors is also indicated.

### SB transposed anti-CCR5 and CXCR4 siRNAs confer HIV-1 resistance

**Figure 3:**
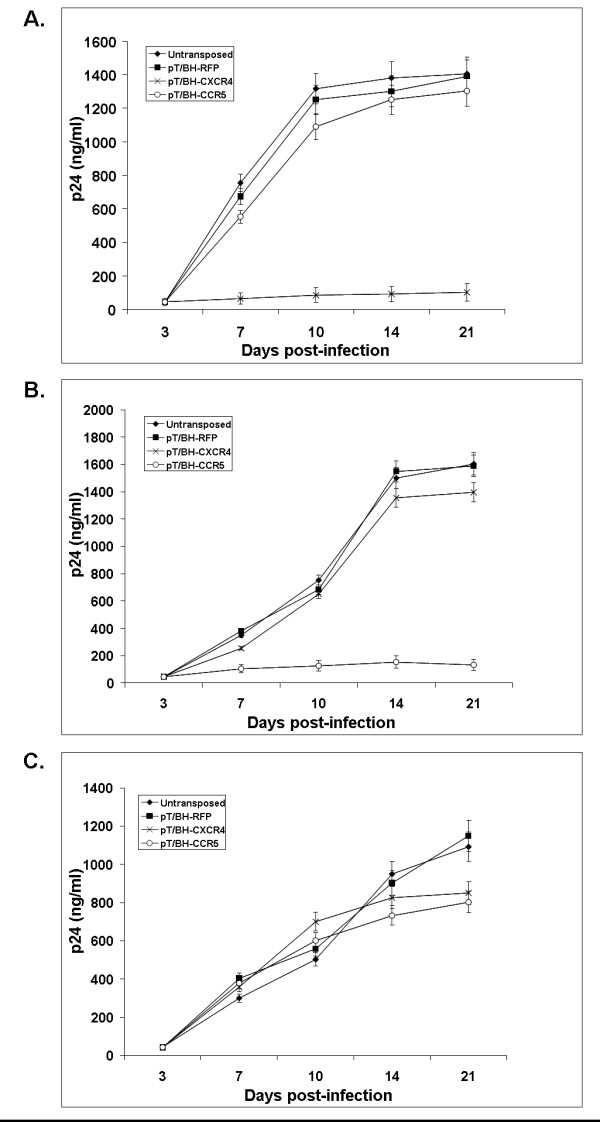
**HIV-1 challenge of siRNA transposed GHOST-R3/X4/R5 cells**. To determine viral resistance, siRNA transposed transgenic cells were challenged with HIV-1 NL4.3 (CXCR4 tropic virus), HIV-1 BaL (CCR5 tropic virus) or HIV-1 89.6 (dual tropic virus) viruses at an MOI of 0.01. On various days post-infection, cell culture supernatants were collected and analyzed for p24 antigen levels by ELISA to determine the levels of viral inhibition. Untransposed (◆), control RFP transposed (■), CXCR4 siRNA transposed (×) or CCR5 siRNA transposed (○). Panel A – NL4.3, Panel B – BaL, Panel C – 89.6.

To determine if down regulation of CCR5 and CXCR4 coreceptors conferred viral resistance, siRNA transgenic GHOST-R3/X4/R5 cells were challenged with X4-tropic (NL4-3), R5-tropic (BaL-1) and dual coreceptor tropic HIV-1 89.6 strain. Antigen ELISAs to detect viral p24 in culture supernatants were performed on various days post-infection up to three weeks (Figure 3). When challenged with X4-tropic HIV-1 NL4.3, GHOST-R3/X4/R5 cells expressing CXCR4 siRNA showed a 10 fold decrease in virus production as compared to control non-transgenic cells on day 10 post-infection. The level of viral inhibition reached upto 14 fold through day 21 post-infection. In contrast CCR5 siRNA expressing GHOST-R3/X4/R5 cells failed to show any inhibition of virus production against X4 tropic HIV-1 NL4.3. Viral challenge of GHOST-R3/X4/R5 cells expressing CCR5 siRNA with the R5-tropic HIV-1 BaL resulted in an 8 fold reduction in virus production on day 10 post-infection, which doubled to 16 fold on days 14 and 21 post-infection. GHOST-R3/X4/R5 cells expressing CXCR4 siRNA served as a negative control as they showed similar levels of infection seen in control non-transgenic cells with the R5-tropic virus challenge. In dual-tropic HIV-1 89.6 viral challenges, neither of the individual CXCR4 siRNA or CCR5 siRNA expressing GHOST-R3/X4/R5 cells showed significant protection as expected since the challenge virus could use either of the coreceptors. However there was a moderate decrease in the virus production on day 21 as compared to unmanipulated cells. Cells transposed with SB control construct without anti-HIV transgenes showed similar levels of infection as the unmanipulated cells for all three HIV-1 strains. We also challenged SB transposed MAGI-CCR5 and MAGI-CXCR4 cells with R5 or X4 tropic viral strains respectively and found similar levels of resistance (data not shown). These data collectively showed that the respective SB system delivered siRNAs are functional and mediate viral resistance.

## Methods

### Construction of CCR5 and CXCR4 shRNA expressing SB constructs

The Sleeping Beauty transposon vector pT/BH plasmid was obtained from Dr. Perry Hackett (University of Minnesota). The vector plasmid contains a multiple cloning site (MCS) flanked by a left and right inverted/direct repeat (IR/DR) elements [27,28]. Based on our previous data two well characterized and effective CCR5 and CXCR4 shRNAs were chosen for incorporating into the SB system plasmid [29]. The CCR5 siRNA target sequence is 5'-GUGUCAAGUCCAAUCUAUG-3' whereas the CXCR4 siRNA target sequence is 5'-GAGUCUGAGUCUUCAAGUU-3'. The CXCR4 or CCR5 shRNA DNA cassette was generated by PCR using published protocol [30]. In brief, PCR was done using U6 or H1 forward primer and a reverse primer containing 3'end homologous region of U6 or H1 promoter fused with CXCR4 or CCR5 shRNA sequence. The resulting PCR product was cloned into a Topo vector pCR8GW (Invitrogen, CA). A BglII site was engineered at the 5'end of forward and reverse primers. pT/BH was the transposon vector plasmid used into which a CMV driven RFP, IRES driven neomycin resistance gene and a SV40 polyadenylation signal containing cassette was cloned at the EcoRV site to derive the control RFP SB plasmid. To generate this pIRESneoRFP cassette, RFP gene was cloned as a BamHI – NotI fragment from pDsRed-N2 (Clontech, CA) into pIRESneo3 (Clontech, CA). A U6 promoter driven CXCR4 shRNA or H1 promoter driven CCR5 shRNA DNA cassette was cloned in parallel as a BglII-BglII fragment in the pT/BH plasmid at BamHI site to get pT/BH-U6CXCR4 or pT/BH-H1CCR5. The CMV-RFP-IRES-neo-SV40pA cassette was released as NruI-BstZ17I fragment and cloned at EcoRV site of pT/BH-U6CXCR4 or pT/BH-H1CCR5 plasmid to get pT/BH-U6CXCR4-CMV-RFP-IRES-neo or pT/BH-H1CCR5-CMV-RFP-IRES-neo. A hyperactive transposase expressing plasmid pHSB5, obtained from Dr. Mark Kay (Stanford University) was used to transpose the SB constructs [8]. A schematic representation of SB constructs and transposase plasmid are shown in Figure 1.

### Cell culture and transfection

Respective coreceptor expressing MAGI-CCR5 and MAGI-CXCR4 cell lines were obtained from the NIH AIDS Reagent Program and maintained in Dulbecco's Modified Eagle Medium (DMEM) supplemented with 10% FBS, 500 μg/ml G418, 100 μg/ml hygromycin and 1 μg/ml puromycin. Similar culturing conditions were used for GHOST-R3/X4/R5 cells with G418 concentration being 200 μg/ml [23-25]. Cells were transfected with respective SB plasmids using Lipofectamine 2000 (Invitrogen, CA) as we previously described [31].

### FACS analysis and sorting

To enrich for transgenic cells, the SB transfected cells were subjected to FACS sorting based on RFP expression. The sorted cells were cultured for 4 weeks and analyzed by FACS to determine the cell surface down regulation by the respective siRNAs as described [32]. Briefly, the transfected or untransfected control cells were washed in PBS and resuspended in FACS buffer. FITC conjugated anti-CXCR4 or anti-CCR5 antibody was added to the cells and incubated for 30 minutes at 4°C. Cells were then washed and resuspended in PBS for FACS which was done using a Coulter EPICS-XL MCL (Coulter Corporation, FL) machine and analysed with EXPO32 ADC software.

### HIV-1 challenge of siRNA transposed cells

To determine viral resistance conferred by the down regulation of CCR5 and CXCR4 coreceptors, siRNA transposed or non-transposed cells were subjected to viral challenge with HIV-1 BaL (CCR5-tropic), HIV-1 NL4.3 (CXCR4-tropic) or HIV-1 89.6 (Dual-tropic) viral strains. The HIV-1 viral strains were obtained from the AIDS Research and Reference Reagent program, Division of AIDS, National Institute of Allergy and Infectious Diseases. Briefly, 0.5 × 10^6 ^transgenic GHOST-X4/R3/R5, MAGI-CXCR4 or MAGI-CCR5 cells in 6 well plates were washed and exposed to virus at an MOI of 0.01 in the presence of polybrene (4 μg/ml). Virus was allowed to adsorb for 2 hours at 37°C. Cells were then washed twice with PBS and 2 ml of complete DMEM was added [21,33]. Culture supernatants collected at different days post-challenge were assayed for p24 antigen by ELISA (Beckman-Coulter, CA).

### Transposed gene integration analysis

To verify the stable transposition of the siRNA containing genes in the RFP expressing cell lines, the genomic DNA was isolated and subjected to Splinkerette PCR using a published protocol [26]. Transposed cell genomic DNA was digested with Sau3AI (for left IR/DR junctional analysis) or NlaIII (for right IR/DR junction analysis). Splinkerretes were generated by heating equimolar amounts of long primerette (5'-CCTCCACTACGACTCACTGAAGGGCAAGCAGTCCTAACAACCATG-3') with the respective splink to 80°C and cooling it to room temperature. Splink BglII (5'-GATCCATGGTTGTTAGGACCTGGAGGGGAAATCAATCCCCT-3', 5'-phosphate) was used for left IR/DR and splink SphI (5'-GTTGTTAGGACTGCTTGGAGGGGAAAATCAATCAATCCCCT-3', 5'-phosphate) was used for right IR/DR. The splinkerretes were then ligated to the respective digested genomic DNA ends. Ligation was performed with 7.5 μM of splinkerette and 25 ng/μl of genomic DNA with T4 DNA ligase (Fermentas Inc, MD). Primary PCR was done using the ligation reaction as template with primerette short (5'-CCTCCACTACGACTCACTGAAGGGC-3') in conjunction with either long IR/DR (L2) (5'-CTGGAATTTTCCCAAGCTGTTTAAAGGCACAGTCAAC-3') for IR/DR (L) or long IR/DR (R) (5'-GCTTGTGGAGGCTACTCGAAATGTTTGACC-3') for IR/DR (R). Primary PCR was done with 10 cycles of 95°C for 5 sec and 70°C (-0.5°C per cycle) for 2 min followed by 20 cycles of 95°C for 5 sec and 65°C for 2 min. Nested PCR was done by using 1/250 dilution of primary PCR product within the secondary PCR reaction. The second PCR was done using primerette-nested (5'-GGGCAAGCAGTCCTAACAACCATG-3') in conjunction with new L1 (5'-GACTTGTGTCATGCACAAAGTAGATGTCC-3') for IR/DR (L) or IR/DR (R) KJC1 (5'-CCACTGGGAATGTGATGAAAGAAATAAAAGC-3') for IR/DR (R). Nested PCR was done with 30 cycles of 95°C for 5 sec, 61°C for 30 sec and 70°C for 90 sec. Both primary and nested PCR included a hot-start at 95°C for 1 min and a final extension of 70°C for 10 min. Oligonucleotides used for this assay were obtained from IDT (San Jose, CA). The PCR products were cloned using a Topo cloning kit (Invitrogen, CA) and sequenced for the junctional region. The sequencing was done by Laragen (Los Angeles).

## Discussion

As a first step towards exploiting a non-viral gene transfer system for HIV gene therapy, here we have shown that SB transposon system can be utilized for deriving stably gene modified cells that display HIV resistance. To achieve this goal, we employed siRNAs with proven efficacy to down regulate expression of the essential HIV-1 coreceptors CCR5 and CXCR4 with a consequent viral resistance phenotype. To our knowledge this is the first report describing gene transfer for viral resistance using a transposon system.

GHOST-R3/X4/R5 cells constitutively expressing both CCR5 and CXCR4 coreceptors were used for SB mediated siRNA gene transfer in these proofs of concept studies. Since the general gene transfer efficiency is low relative to that typically obtained with lentiviral vectors [7,33,34], transfected cells were enriched by FACS sorting to evaluate the effectiveness of the stably integrated siRNA transgenes. Our results have shown that transgenic cells could be cultured indefinitely with stable expression of the transposed genes. FACS analysis of the siRNA modified cells showed consistent down regulation of the respective receptors CCR5 and CXCR4 amounting up to a 94% down regulation whereas cells transposed with control SB construct lacking siRNA transgenes showed normal levels of coreceptor expression similar to unmanipulated cells. Thus, down regulation of the respective targeted coreceptors established that siRNA transgenes are functional in a SB transposon system. As determined in the viral challenge experiments, siRNA transgenic cells also showed HIV resistance. With regard to individual siRNAs, GHOST-R3/X4/R5 cells transposed with CCR5 siRNA were found to be resistant to R5 HIV-1 viral challenge, whereas the cells transposed with CXCR4 siRNA were resistant to X4 HIV-1 viral challenge thus confirming the specificity of the respective siRNAs in mediating viral resistance. As expected, no significant protection could be seen from a dual tropic viral challenge of either of the individual siRNA gene modified cells since this viral strain could use either of the coreceptors for cellular entry.

To further confirm stable gene transposition of the siRNA genes, we also mapped the integration sites of the SB transposon in respective transfected cells and found that these representative cell clones harbored the transgenes in different chromosomes namely 5, 6, 7, 10, 12, 15, 17 and 20. Previous studies mapped numerous SB-mediated integration sites in cultured and primary cells and found no chromosomal preference for insertion [35,36]. Consistent with this observation, the above clones transposed with siRNAs also represent random transposition events.

The non-viral nature of the SB system offers some advantages over the more common retro and lentiviral mediated gene transfer [37]. Among these are that no viral sequences are involved thus minimizing insertion transcriptional activation of cellular genes and risk of generation of replication competent viruses during vector production. However, the gene transfer efficiency with the SB system remains sub-optimal compared to the viral vector systems [9]. Future improvements in the SB system are necessary to achieve higher gene transfer efficiency to be clinically practical [6,9].

Although shown to be effective in conferring HIV resistance to cultured cells, the present SB system needs to be further evaluated in a hematopoietic stem cell setting using CD34 progenitor cells with a high efficiency of gene transfer to be clinically useful as shown with lentivirus vectors [3,4]. Even if high enough efficiency gene transfer is not achievable with this system in the near future, other innovative approaches are possible that may show clinical utility. For example, currently human embryonic stem (hESC) cells show great promise in developing novel therapies [38,39]. The hESC have already been shown to be amenable to gene transfer with SB transposon system, and it is now routine to derive hematopoietic CD34 cells from hESC as shown by us and others [40-44]. One can envisage that hESC can be transposed with anti-HIV siRNAs using SB system and high expressing cell clones could be derived. From these transgenic hESC clones, unlimited numbers of siRNA expressing CD34 cells could be derived for HIV gene and cell therapies. Such experiments are currently underway in our laboratory.

## Conclusion

SB gene transposon system can be used to deliver siRNA genes against HIV-1 coreceptors CCR5 and CXCR4 for stable gene expression. The siRNA genes are able to downregulate the respective coreceptor expression on the cell surface and thus confer resistance against HIV-1 infection by restricting viral entry. These studies have demonstrated for the first time the utility of the non-viral SB system to derive viral resistant cells and paved the way for the use of this system for HIV gene therapy studies.

## Competing interests

The authors declare that they have no competing interests.

## Authors' contributions

MT derived the experimental data and RA was responsible for the conception and overall implementation of the project. All authors read and approved the final manuscript.
